# Surgical Insights for the Management of Variant Histology in Renal Cell Carcinoma

**DOI:** 10.1590/S1677-5538.IBJU.2020.0778

**Published:** 2021-01-10

**Authors:** Mauro Antonio Dispagna, Michael Daneshvar, Gennady Bratslavsky

**Affiliations:** 1 SUNY Upstate Medical University College of Medicine SyracuseNY United States College of Medicine, SUNY Upstate Medical University, Syracuse, NY, United States; 2 SUNY Upstate Medical University Department of Urology SyracuseNY United States Department of Urology, SUNY Upstate Medical University, Syracuse, NY, United States

**Keywords:** Carcinoma, Renal Cell, Histology, Surgical Procedures, Operative

## Abstract

**Purpose:**

To review the current literature regarding variant (non-clear) histology of renal cell carcinoma (RCC) and the clinical management of these renal tumors.

**Material and Methods:**

A PubMed database search was performed in May 2020 focusing on variant RCC, its diagnosis and associated syndromes, tumor characteristics, and options for management.

**Results:**

A broad range of pathological, clinical and diagnostic characteristics amongst non-ccRCC variants were found to have an impact on the overall management of these tumors. The imaging modalities, frequency of surveillance, and timing for intervention were found to be dependent on the type of genetic alterations, type of histology, and tumor growth rates. The timing and type of surgery as well as the systemic therapy are tailored to the specific tumor type and patient.

**Conclusion:**

The findings of this review suggest that clinical management should be considered and adjusted for patients with non-ccRCC histological variants based on tumor subtype and genetic alterations.

## INTRODUCTION

Renal cell carcinoma (RCC) is the most common type of primary tumor of the kidney in adults and accounts for nearly 90% of all renal malignancies (1). The incidence of RCC continues to increase by approximately 2-3% each year as a result of the increased utilization of cross-sectional imaging (2). In the United States, there are more than 70.000 new cases of RCC diagnosed each year with approximately 15.000 deaths. Although the majority of cases of RCC are sporadic, approximately 4% of them have a genetic component (3).

RCC can be divided into histological subtypes based on molecular and genetic characteristics. The majority of RCC are clear cell RCC (70-90%). The majority of clear-cell RCC (ccRCC) are associated with a mutation or epigenetic silencing of the von Hippel-Lindau (VHL) tumor suppressor gene on chromosome 3 (4). Non-clear cell RCC (ncRCC) encompasses a group of renal malignancies with varying histological and molecular features that affect tumor behavior and ultimately, clinical management.

The variant histologic subtypes of non-clear cell RCC include: Papillary Type 1 and 2, Chromophobe, Transcription factor E3 (TFE3), Oncocytic, Clear-cell/Chromophobe and sometimes high-grade clear cell (when the histologic appearance is so dedifferentiated that clear cell component is not readily appreciated (Table-1). Oncocytoma and Angiomyolipoma are often included in a group of renal “non-clear” neoplasm but are almost always benign. Multiple studies have shown significant differences between the metastatic potential, growth kinetics and structural and histologic attributes (such as peritumoral pseudocapsule) of ncRCC histological variants (5–7). The diagnosis of ncRCC has implications on the surveillance, timing of surgery, surgical modality and potential need for systemic therapy.

**Table 1:** Renal cell carcinomas and genetic correlates (4–8), (10–12).

Mutated Gene	Chromosome	Tumor Type
von Hippel-Lindau (VHL)	3	Clear Cell
MET-proto oncogene	7	Papillary Type 1
Fumarate Dehydrogenase (FH)	1	Papillary Type 2
Folliculin (FLCN)	17	Chromophobe; Oncocytoma; Hybrid;
Tuberous sclerosis complex 1 (TSC1)	9	
Tuberous sclerosis complex 2 (TSC2)	16	Angiomyolipoma
Xp11.2 translocation	X and 11	Transcription factor E3 (TFE3) RCC
Succinate-dehydrogenase	1	Oncocytic
Phosphatase tensin homolog (PTEN)	10	Clear-cell/Chromophobe
BRCA1 associated protein-1 (BAP-1)	3	High grade, Clear-cell

The most commonly occurring ncRCC variants include papillary RCC (10-15%) and chromophobe RCC (3-4%) (1, 8). Papillary RCC (pRCC) can be further classified as type 1 or type 2 tumors based on differing histological features and genetic findings. Mutations at the MET proto-oncogene on chromosome 7 have been associated with the development of Type 1 pRCC (9). Papillary type 2 is the most common histological subtype of RCC that occurs in hereditary leiomyomatosis and renal cell cancer (HLRCC) a condition in which the fumarate hydratase (FH) gene is mutated. Another rare autosomal dominant syndrome, Birt-Hogg-Dubé disease (BHD), occurs due to mutations of the Folliculin gene (FLCN) on chromosome 17 (10). Patients with mutations of the FLCN gene are more likely to develop chromophobe, oncocytoma and oncocytic-chromophobe hybrids of RCC (11). Other less frequently occurring subtypes of non-clear cell histologies include: succinate dehydrogenase-deficient RCC (SDHB, SDHC, SDHD) and MiT family translocation RCC (TFE3, TFEB). Additionally, mutations in PTEN and BAP-1 genes have been correlated with clear-chromophobe and clear, high-grade variants of non-clear RCC, respectively (2, 11). Angiomyolipomas are almost invariably benign and are seen in tuberous sclerosis syndrome, associated with mutations in TSC1 and TSC2 genes. Table-1 summarizes various tumor types and associated mutations of their genes.

Over the last several decades, genetic alterations have been identified in rare RCC subtypes that ultimately affect the clinical workup and management of patients. Genetic testing and biopsy of the mass may be useful in identifying a variety of ncRCC subtypes which may help drive different therapeutic or interventional treatments. Physicians should maintain a high-level of suspicion for ncRCC not only on radiographic findings but also based on patient demographics (3). For example, a 2019 retrospective analysis by Batai et al. of 405.073 cases of RCC from the National Cancer database (NCDB) and 9.751 cases from Arizona Cancer registry (ACR) found that Hispanic, American Indians and Alaska natives have a younger age of onset and higher prevalence of ccRCC histological subtype when compared to their non-Hispanic white counterparts (13). Additionally, Daugherty et al. found that chromophobe RCC is the most common type of non-clear cell RCC in young patients, and especially young women (14). Patient demographics coupled with additional risk factors such as: cigarette smoking, obesity and hypertension (15) may prompt physicians to perform a confirmatory test of the renal mass- such as a core needle biopsy to identify the histological subtype of the tumor. Additionally, some ncRCCs are so aggressive that even small tumors may present with metastatic disease. For example, in one study of papillary type 2 RCC, four of seven patients with 2.0 to 6.7cm T1 tumors had spread to regional lymph nodes or had metastases at nephrectomy (12). This rate of metastatic RCC is much greater than one would expect or observe in patients with clear cell RCC (5). Once the subtype of RCC has been identified, the next challenge is choosing the correct treatment plan. To date, data for the treatment and management of ncRCC subtypes is sparse (unless the disease is localized). This review discusses the workup, evaluation, management and follow-up of patients with variant histologic subtypes of RCC with guidance for clinicians when ncRCC is suspected.

### Imaging

Based on the most recent American Urological Association (AUA) guidelines, the ideal imaging modality for the diagnosis and staging of renal masses is pre and post contrast-enhanced abdominal imaging. This includes computed-tomography (CT), magnetic resonance imaging (MRI) and ultrasound (US). Multi-phasic CT with contrast remains the first line modality in evaluating renal masses (16). However, certain limitations, such as detecting hypo-enhancing lesions (17, 18) (e.g. papillary RCC, AML), require the use of other modalities, such as contrast-enhanced ultrasound (CEUS) or MRI. Thaiss et al. describe the use of CEUS and acoustic radiation force impulse (ARFI) elastography to characterize CT-indeterminate renal masses (under <4cm). The authors identified that oncocytoma and ccRCC have higher peak intensities than chromophobe and papillary when using CEUS (19). These findings suggest that some renal masses such as, papillary or chromophobe, are not easily identified on standard US, therefore surgeons who choose CEUS for active surveillance of renal masses in their patients must beware of such lesions. A retrospective study performed by Yenice et al. in 2020, found that the use of MRI for characterizing cystic renal masses resulted in the upgrading and downgrading of Bosniak classification of the masses and ultimately, affected the surgical management of these patients (20). One study claims that multiphase- MRI is highly sensitive at differentiating the enhancement patterns of ccRCC, pRCC and chRCC, suggesting MRI should be used for the management of ncRCC (21).

The future of renal imaging, however, is currently evolving with the use of machines and artificial intelligence (AI). Kocak et al. used machine learning-based quantitative CT texture analysis (qCT-TA) and discovered that these machine algorithms can reliably differentiate between ccRCC and ncRCC renal masses with high specificity (22). Another study that used a quantitative computer-aided diagnostic (CAD) algorithm, also found significant differences in peak attenuation that allowed for discrimination of ccRCC and non-ccRCC from four-phase multidetector CT (23). It is crucial for surgeons to correctly identify renal lesions as the growth rates and metastatic potential of renal tumors vary significantly and will directly impact the timing of surgery.

## TREATMENT OPTIONS

### Active surveillance

Active surveillance is a safe initial option for the management of renal masses, especially those that are <2cm in size or when the risk of intervention outweighs the benefits of treatment (24). The current American Urological Association (AUA) guidelines emphasize the importance of a baseline assessment of tumor, patient and treatment related risk factors prior to the decision to pursue active surveillance. During active surveillance, a strict imaging protocol is followed in order to monitor the potential growth of the renal mass (7). This includes but is not limited to renal imaging every six months. Traditionally, tumor size or growth rates have been utilized for surgical decision making in patients with renal masses. In fact, the American Joint Committee on Cancer (AJCC) uses tumor size in predicting cancer-specific survival (CSS) rates. Although these measures are appropriate in the majority of cases, the influence of varying histology on metastatic potential and cancer-specific survival cannot be overlooked. Multiple studies have shown significant differences in metastatic and cancer specific survival rates amongst the varying histological subtypes of RCC (25–27). Daugherty et al. described small-renal masses to include the metastatic potential of the lesions based on their histologic subtypes (5). They described that size alone did not predict the metastatic potential, as they found significant differences in metastatic rates between clear-cell and non-clear cell variants of RCC. Based on their data from the SEER-18 registries database, clear-cell and papillary histological subtypes crossed a 5% metastatic rate at a size of 6cm, whereas the chromophobe RCC crossed this same 5% rate at a size of 10cm. Therefore, active surveillance protocols, patient counseling and timing of surgery should be more frequent and rigorous for clear cell and papillary type 1 than chromophobe RCC. Another study of 41 patients with renal masses followed for a mean duration of 29 months found no statistically significant difference between growth rates of biopsy proven oncocytoma (mean 0.52cm/yr) and clear-cell RCC (mean 0.71cm/year) (28). Additionally, in 2011 Jewett et al. found that biopsy proven malignant and benign small-renal masses may grow rapidly, grow slowly, not grow or even regress (7). The differences observed in growth kinetics between histological variants of RCC subtypes suggests physicians should adjust surveillance frequencies based on tumor histology. In addition to modifying pre-surgical procedures for management of RCC histological variants, surgical techniques also should be tailored for management of ncRCC.

### Partial Nephrectomy (PN)

The most common surgical interventions for removal of renal masses are partial nephrectomy (PN) and radical nephrectomy (RN) (29). In the past two decades, several studies have demonstrated the feasibility of using aggressive PN in patients with hereditary and multifocal renal cancers (30–33). Gupta et al. described the use of PN in treating hereditary renal cancers as oncologically safe. They found similar metastasis-free survival and overall survival rates to that of sporadic RCC cases treated with PN (32). Additionally, a retrospective study of 128 patients with bilateral renal masses treated with nephron-sparing surgery, found the cancer-specific survival (CSS) rates, at a minimum of 10-year follow-up, to be up to 97% (33). The use of minimally invasive techniques such as robotic assisted PN for multifocal tumors was also shown to be surgically feasible in one study that successfully removed 24 tumors in 9 patients without the need for hilar clamping (30). Finally, Fadahunsi et al. found more than 80% of the perioperative renal function following multifocal PN to be preserved (31). Conversely, a large meta-analysis by Zhang et al. suggests that the use of PN in treating ncRCC histological variants is not an oncologically safe choice. They compiled 13 studies with over 47.000 patients and evaluated the relationship between various clinical variables and the rate of positive surgical margins (PSM) for patients with RCC undergoing PN. They found a statistically significant association between PSM and patients with ncRCC (pooled OR=0.78; 95% CI: 0.72-0.84; P <0.001), as well as non-white race (pooled OR=0.90; 95% CI: 0.82-0.99; P=0.026) (34). Although the results of these studies indicate the use of PN in treating multifocal disease and those with familial renal cancer syndromes to be a reasonable option, surgeons must be extra cautious and vigilant of the possibility of positive surgical margins when treating ncRCC tumors with PN.

### Enucleation

One specific form of PN includes a technique where a tumor is enucleated from the parenchyma of the kidney (35). The enucleation includes a careful dissection around the tumor using the tumor-parenchymal interface as an anatomic guide for resection. A large retrospective analysis performed by Carini et al. demonstrated that simple enucleation of pT1a histologically proven RCC (including 7.8% papillary and 6.5% chromophobe) to be an oncologically safe procedure with 5 and 10-year CSS rates of 96.7% and 94.7%, respectively (36). Perhaps more impressive is the fact that none of the 232 patients had local recurrences of cancer at the level of the enucleation bed. Another study performed by Carini et al. on the safety of enucleation of RCC between 4 and 7cm, again demonstrated the efficacy of the procedure. This study found similar cancer-specific survival rates to radical nephrectomy, with no significant risk of local recurrence when compared to partial nephrectomy for masses under 4cm (37). In addition, they found the cancer-specific survival rates of treating pT1b RCC with enucleation to be 95.7%. Interestingly, this study also found pT1b and pT3a cancer-specific survival rates to be 83.3% and 58.3%, respectively, suggesting tumor size to be a determinant of enucleation success. However, size alone may not be the only factor in predicting successful enucleation of renal masses. Enucleation success may also depend on the tumors pattern of pseudocapsule (PC) invasion, which varies depending on the histology of the renal mass.

In a study of 160 pT1 renal tumors, Jacob et al. found significant differences between RCC subtypes PC characteristics and invasion. In that study, they found complete PC in 77% of clear cell tumors, 74% of papillary, 28% of chromophobe, and 4% of oncocytomas and partial PC in nearly 44% of the chromophobe and 56% of oncocytoma subtypes. Importantly, they showed that PC invasion was predictable based on tumor histology, with papillary RCC having the highest rate of invasion through the capsule at 30% followed by clear-cell RCCs at 8% and none of the chromophobe and oncocytomas RCC showing complete PC invasion (6). Minnervini et al. also demonstrated that 121/127 (95%) of renal tumors had a well-defined PC and that 24/121 (19.8%) had a complete invasion of that capsule, with significant differences seen between variant histological subtypes of RCC. They found that papillary RCC had a much higher likelihood of PC invasion with an odds ratio of 6.57 of complete PC invasion when compared to clear-cell RCC (38). Therefore, with careful preoperative determination of tumor type and histology, enucleation can be a feasible operative technique for the removal of some renal masses but certainly not all. The appreciation of variability in pseudocapsular integrity is an important surgical consideration and may explain Zhang's et al. findings of a statistically significant association between PSM and patients with ncRCC (34).

### Surgical management in metastatic disease: cytoreductive nephrectomy

In the setting of metastatic RCC, cytoreductive nephrectomy can be performed, but its benefits in treating ncRCC histological variants is controversial and beyond the scope of this review. In 2007, Kassouf et al. evaluated the use of cytoreductive nephrectomy in patients with metastatic RCC of both clear-cell and non-clear cell variants and found significant differences between the two groups. They determined that patients with metastatic ncRCC had a higher incidence of sarcomatoid features and a worse prognosis when compared to patients with metastatic ccRCC. Patients treated with cytoreductive nephrectomy for metastatic ncRCC had a median disease specific survival of 9.7 months and patients with metastatic ccRCC had a median disease survival of 20.3 months (39). Shuch et al. then reviewed the role of cytoreductive nephrectomy in patients with sarcomatoid features and found that although these patients presented with similar clinical characteristics those with sarcomatoid features had a higher incidence of having non-clear cell histology than patients without sarcomatoid features. Notably, the median survival of patients with sarcomatoid features was 4.9 months and those with no sarcomatoid histology was 17.7 months (9). When cytoreductive nephrectomy is used in the treatment of metastatic RCC, the histological features of the tumor can impact the effectiveness of this treatment method.

Most recently, CARMENA trial questioned the role of cytoreductive nephrectomy in patients with metastatic RCC (40). Interestingly, presence of ncRCC places patients into the unfavorable or high-risk group, thus likely limiting the role of CN in this patient population. Nevertheless, with new therapies, combinations, and trials on horizon, the CN may still have a role in well selected patients.

### Retroperitoneal Lymph Node Dissection (RPLND)

Another careful consideration in the treatment of variant histology RCC is whether or not conducting a retroperitoneal lymph node dissection is necessary or beneficial (RPLND). In 2016, Gershman et al. identified 305 patients treated with cytoreductive nephrectomy for M1 RCC and compared the association between RPLND and cancer-specific mortality as well as all-cause mortality. They found no differences between cancer-specific or all-cause mortality in patients undergoing RPLND for metastatic RCC (pN1) versus those patients who did not undergo RPLND (41). Therefore, suggesting that RPLND in the treatment of metastatic RCC is not associated with improved oncological outcomes. Additionally, prior randomized trial by Blom et al. found no benefit of RPLND for small renal masses (42). However, in some cases, for example, those with FH driven RCC (i.e. HLRCC) renal tumors can metastasize to lymph nodes before they reach 1cm in the largest dimension (12). In these select patients, RPLND may be curative and represents an appropriate surgical option.

### Systemic therapy

Similar to the surgical approaches to RCC, systemic therapy options should be adjusted based on tumor and patient characteristics. The majority of clinical trials for the use of systemic therapy in RCC have been focused on clear-cell histology. Available agents for treating metastatic RCC include: mammalian target of rapamycin (mTOR) inhibitors (e.g. everolimus and temsirolimus), vascular endothelial growth factor (VEGF) inhibitors (e.g. sunitinib, lenvatinib, bevacizumab), programmed cell death protein 1 (PD-1) checkpoint inhibitors (e.g. nivolumab and pembrolizumab), programmed cell death ligand 1 (PD-L1) checkpoint inhibitors (atezolizumab), anticytotoxic T lymphocyte-associated protein 4 (CTLA-4) antibodies (ipilimumab). Early data suggest that targeted immunotherapy with PD-1 and PD-L1 inhibitors could have a positive effect in patients with metastatic non-clear cell variant histologies (43, 44). Although there is currently no standard treatment of metastatic ncRCC, current ongoing clinical trials are investigating the role of CPI (checkpoint inhibitors), VEGF and mTOR inhibitors. Perhaps, those tumors with a high TMB (tumor mutational burden) and MSI (microsatellite instability) could have a robust response to CPI.

## CONCLUSIONS

Clinicians must remain vigilant for variant histology amongst renal tumors. Clinical management should be modified based on genetics and tumor histological characteristics. Active surveillance frequency and diagnostic imaging modalities must be adjusted in management of ncRCC as growth kinetics are often different from ccRCC. The observed discrepancies between metastatic potential of renal masses, metastasis to lymph nodes and characteristics of PC invasion may affect the timing of surgery, surgical technique, and acceptance of surveillance of these masses with variant histology. Finally, systemic therapy should take into consideration the histologic findings of each tumor as genetic discoveries have the potential to direct therapeutic targeting.
